# Changes in the mechanical properties of the thigh and lower leg muscle-tendon units during the early follicular and early luteal phases

**DOI:** 10.3389/fspor.2024.1323598

**Published:** 2024-03-26

**Authors:** Rina Saito, Mayuu Shagawa, Yuzuka Sugimoto, Tomoki Hirai, Koyo Kato, Chie Sekine, Hirotake Yokota, Ryo Hirabayashi, Tomonobu Ishigaki, Hiroshi Akuzawa, Ryoya Togashi, Yuki Yamada, Haruki Osanami, Mutsuaki Edama

**Affiliations:** Institute for Human Movement and Medical Sciences, Niigata University of Health and Welfare, Niigata, Japan

**Keywords:** menstrual cycle, mechanical properties, stiffness, estradiol, muscle-tendon complex

## Abstract

**Background:**

This study aimed to determine changes in the muscle and tendon stiffness of the thigh and lower leg muscle-tendon units during the early follicular and early luteal phases, and check for possible relations between muscle and tendon stiffness in each phase.

**Methods:**

The sample consisted of 15 female university students with regular menstrual cycles. The basal body temperature method, ovulation kit, and salivary estradiol concentration measurement were used to estimate the early follicular and early luteal phases. A portable digital palpation device measured muscle-tendon stiffness in the early follicular and early luteal phases. The measurement sites were the rectus femoris (RF), vastus medialis (VM), patellar tendon (PT), medial head of gastrocnemius muscle, soleus muscle, and Achilles tendon.

**Results:**

No statistically significant differences in the thigh and lower leg muscle-tendon unit stiffness were seen between the early follicular and early luteal phases. Significant positive correlations were found between the stiffness of the RF and PT (*r* = 0.608, *p* = 0.016) and between the VM and PT (*r* = 0.737, *p* = 0.002) during the early luteal phase.

**Conclusion:**

The present results suggest that the stiffness of leg muscle-tendon units of the anterior thigh and posterior lower leg do not change between the early follicular and early luteal phases and that tendons may be stiffer in those women who have stiffer anterior thigh muscles during the early luteal phase.

## Introduction

The menstrual cycle is regulated by various regular fluctuations in female steroid hormones (1–3). And based on these fluctuations, specifically the gonadotropin-releasing hormone from the hypothalamus, follicle-stimulating hormone, and luteinizing hormone from the anterior pituitary, and estrogen (E2) and progesterone (P4) from the ovary gland (3), it is classified into four phases. These changes in hormonal concentrations may have an effect on the mechanical properties of muscles and tendons.

A previous study investigating the rate of muscle-tendon injuries has reported that the injuries are 88% higher during the late follicular phase when E2 levels are maximal than during the follicular phase. In addition, compared with other phases, muscle tears, strains, spasms, tendon disorders, and tendon ruptures have been shown to occur more than twice as often during the late follicular phase (4). These findings suggest that female hormones may affect the structure of muscles, tendons and ligaments.

A relationship between female hormones and the structure of the muscle-tendon unit has been reported regarding the E2 receptor expression in myofibers (5) and tendons (6). E2 has also been found to act on the type I collagen synthesis and fibroblast proliferation, as well as type I collagen degradation by matrix metalloproteases (MMPs) (7). A previous study that treated fibroblasts detached from human thigh fascia with E2, followed by culturing, found decreases in type I collagen and increases in type III collagen and fibrillin, which enhanced the elasticity of the fascia, when E2 concentrations were increased to a level equivalent to that present in humans before ovulation (8). Therefore, multiple pathways exist for the effect of E2 on muscles and tendons, and different tissues appear to be affected in different ways, which suggests that changes in mechanical properties during the menstrual cycle vary by individual.

However, an *in vivo* study using ultrasonography that examined the mechanical properties of human muscle-tendon units during the early follicular, ovulatory, and luteal phases found no significant changes in maximal isometric voluntary contraction or muscle activation levels and tendon properties (maximal elongation and stiffness) of the knee extensors and ankle plantar flexors during the menstrual cycle (9). On the other hand, studies using shear-wave elastography have demonstrated lower muscle stiffness during contraction in the ovulation compared to menstruation phase (10) however no menstrual cycle-induced effects on mechanical properties of the muscle-tendon units (11). As described above, studies have been conducted using ultrasonography and shear-wave elastography, which are considered the golden standards for evaluating muscle and tendon units. On the other hand, recently, several studies have used a noninvasive digital palpation device (MyotonPRO; Myoton AS, Tallinn, Estonia) to evaluate the mechanical properties of human muscle (12–14). The MyotonPro is a non-invasive hand-held, affordable, and easy-to-use myotonometer device aimed at recording the biomechanical and viscoelastic stiffness of myofascial tissues (15, 16). In addition, the short measurement time also has the advantage of allowing measurement of a large number of objects. One of these studies that examined changes in the mechanical properties of the thigh musculature during the early follicular, ovulatory and luteal phases reported increased stiffness of the vastus medialis and semitendinosus during the ovulatory compared with the luteal phase, but no changes in the stiffness of the vastus lateralis or biceps femoris throughout the menstrual cycle (12). A previous study that investigated the mechanical properties of lower leg muscle groups during the early follicular and ovulatory phases found no changes in the stiffness of the peroneus longus, tibialis anterior, or medial head of the gastrocnemius (13, 14). Thus, a sufficient consensus has yet to be reached. Therefore, to determine whether the mechanical properties of the muscle-tendon units change during the same menstrual cycle, it is necessary to examine muscle and tendon separately, site-specific differences, and the relationship between each.

Given this background and assuming that different ovarian hormonal concentrations may affect the E2 receptor of the parallel elastic elements, thereby changing its collagen content and corresponding mechanical resistance, the present study aimed to investigate changes in stiffness of the muscles and tendons of the anterior aspect of the thigh and the posterior aspect of the lower leg during the early follicular and early luteal phases and to determine the existence of correlations between muscular and tendinous stiffness in female university students. We hypothesized that, compared with the early follicular phase, the stiffness of the thigh and lower leg muscles and tendons would not decrease during the ovulatory phase and that correlations would not be found between the stiffness of muscle-tendon units.

## Materials and methods

### Subjects

**Figure 1:**
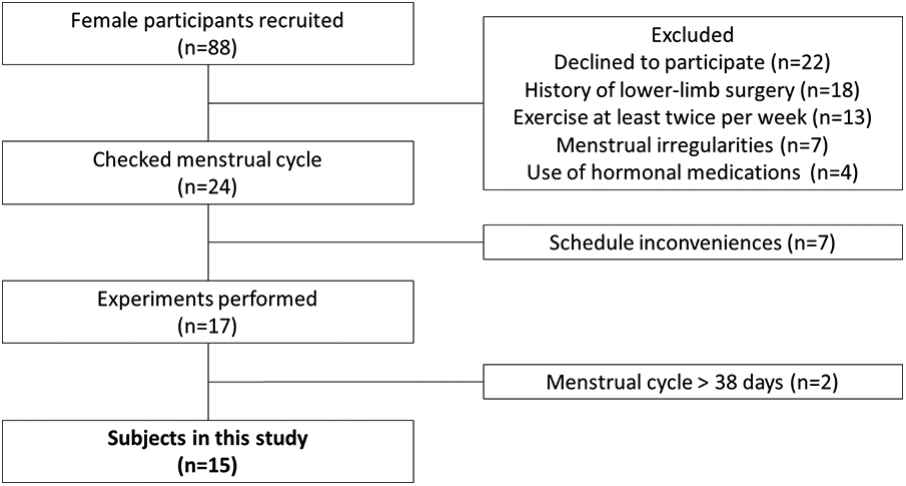
Flowchart for the selection of the female participants.

We conducted a questionnaire survey on 88 female university students. The inclusion criteria were as follows: (1) menstruation approximately 10 times/year, with a menstrual cycle ranging from 25 to 38 days (17); (2) not currently exercising more than twice a week or holding a membership in a designated strength or athletic club (17); (3) no use of oral contraceptives or other hormonal agents within the previous 6 months (18); and (4) no history of doctor-diagnosed disorders or surgery on any part of the lower limb. Of the 88 students, 15 [mean age ± standard deviation (SD), 20 ± 0.5 years; height, 159.2 cm ± 7.1 cm; body mass, 55.9 ± 7.5 kg] met the inclusion criteria and provided written informed consent to participate in the study after receiving a full explanation of all study contents (Figure 1). All study protocols were carried out according to the Declaration of Helsinki after receiving approval from our institutional ethics committee (approval No. 17946).

### Recording of the menstrual cycle

For 1–2 months before the start of the experiment, all participants were asked to measure their basal body temperature every morning after waking up using an electronic basal thermometer (CTEB503l; Citizen Systems, Tokyo, Japan). The date of ovulation was then estimated using an ovulation kit (Doctor's Choice One Step Ovulation Test Clear; Beauty and Health Research, Torrance, CA, USA). When urinary LH was 20 mIU/ml or higher, a red line was displayed on the kit, indicating the start of an LH surge, and about 24 h later was estimated to be the day of ovulation. Using a conditioning management system (ONE TAP SPORTS; Euphoria Corporation, Tokyo), each subject recorded the results of daily basal body temperature, menstruation, and ovulation kits on their own (17–20).

### Timing of measurements

The mechanical properties of the muscle-tendon unit and the concentrations of salivary E2 and P4 were each measured once on the same day during the early follicular and early luteal phases. The measurements in the early follicular phase were recorded on the second to fourth day after menstruation onset, and those in the early luteal phase on the second to fourth day after a positive result from the ovulation kit (17–20). All measurements were conducted from 08:00 to 12:00 to allow for diurnal variations under a room temperature of 20–25°C.

### Measurement methods

#### Concentrations of estradiol and progesterone

The concentrations of E2 and P4 were measured from saliva. Based on previous studies (17–20), the participants were asked to observe the following six points strictly before collecting saliva to help avoid the possibility of influencing E2 and P4 concentrations: (1) refrain from consuming alcohol for at least 12 h prior to measurement; (2) refrain from consuming food for at least 60 min prior to measurement; (3) refrain from toothbrushing at least 45 min prior to measurement; (4) refrain from consuming dairy products for at least 20 min prior to measurement; (5) refrain from consuming beverages with high sugar content, high acid content, or caffeine; and (6) in the case of dental treatment, avoid collecting saliva within 48 h after treatment. All participants were also asked to rinse their mouth before the beginning of the experiment to ensure that no food particles remained in the mouth. To prevent a decrease in E2 and P4 concentrations, saliva was collected at least 10 min after rinsing the mouth using a special straw (Saliva Collection Aid; Salimetrics, State College, PA, USA) and then placed in a saliva collection vessel (Cryovial; Salimetrics) after collection in the mouth for 1 min. Next, all saliva samples were immediately frozen in a freezer at −80°C or colder. E2 concentrations were analyzed by Funakoshi Corporation (Tokyo, Japan) after all samples had been collected. The samples were then thawed at room temperature, mixed immediately by vortexing, centrifuged at 1,500 × g for 15 min, and subjected to analysis by enzyme-linked immunosorbent assay (17β-Estradiol and Salivary Progesterone Enzyme Immunoassay Kits; Salimetrics). The dilution factor was uniformly onefold (undiluted solution) (17–20).

### Mechanical properties of the muscles and tendons

**Figure 2:**
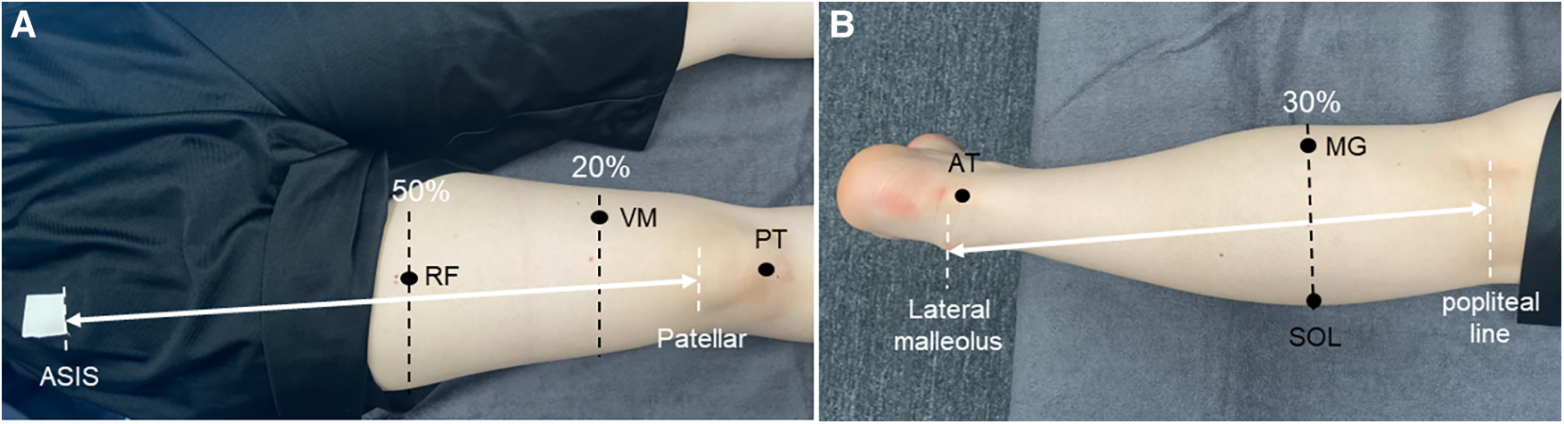
Measurement site for mechanical properties. (**A**) Measurement sites on the thigh. (**B**) Measurement sites on the lower leg. ASIS, anterior superior iliac spine; RF, rectus femoris; VM, vastus medialis; PT, patellar tendon; AT, achilles tendon; MG, medial head of the gastrocnemius; SOL, soleus.

**Figure 3:**
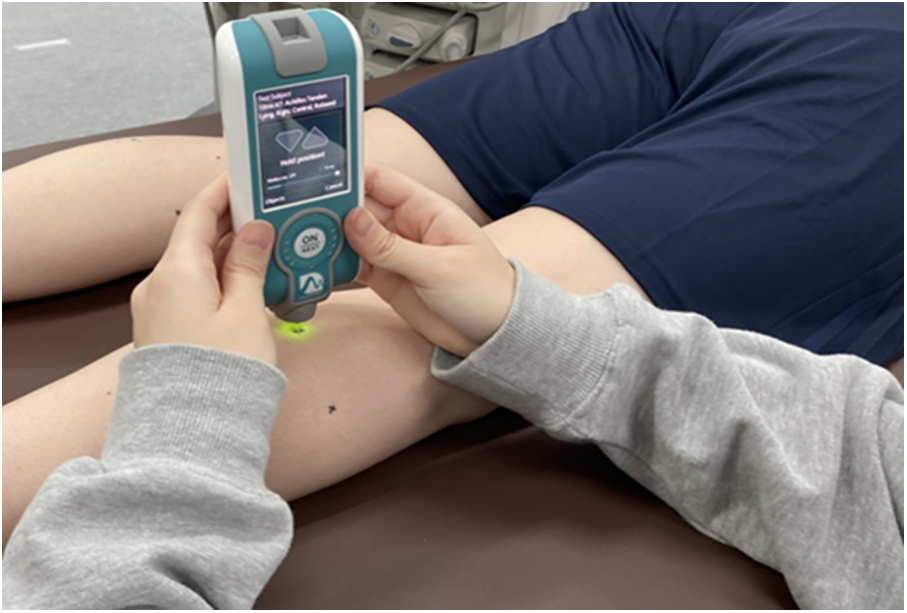
Mechanical property measurement using MyotonPRO.

The stiffness of the muscles and tendons was measured at the following six sites: rectus femoris (RF), vastus medialis (VM), patellar tendon (PT), Medial head of gastrocnemius (MG), soleus (SOL), and Achilles tendon (AT). An ultrasound imaging system (Aplio500; Canon Medical Systems, Tochigi, Japan) and a linear probe (PL0081; Canon Medical Systems) were used to identify all measurement positions, with reference to previous studies (11, 21–23). The RF muscle was identified at half the length from the anterior superior iliac spine (ASIS) to the bottom of the patella (22), the VM at the distal 20% of the length from the ASIS to the bottom of the patella (22), the PT at the midpoint between the patellar apex and tibial rough surface (23), the MG and SOL at the proximal 30% of the length from the orbital skin line to the external capsule (11), and the AT at 3 cm proximal to the tendon attachment site (i.e., the calcaneal tuberosity) (21) (Figure 2). The noninvasive MyotonPRO digital palpation device, which can evaluate the stiffness of muscle on the surface of the skin by making a mark with a pen just above the muscle belly or central part of the tendon, was used to measure the stiffness of the muscles and tendons (Figure 3). The measurement technique used by this device applies five brief mechanical impulses (time, 15 ms; force, 0.4 N) under a steady pre-compression force (0.18 N) of the subcutaneous tissue layer above the evaluated muscle. A device probe (diameter, 3 mm) set perpendicular to the surface of the skin delivers mechanical deformation, and an acceleration sensor connected to the frictionless measurement mechanism detects the damped oscillation produced by the muscle after the brief mechanical impulse. If the Device was rolled more than 10°, a warning notice “Rotate” was displayed. And the device offered the coefficient of variation (CV) for stiffness. The CV described the relative dispersion of the measurement. The CV above 3% was showed in red and re-measured if the CV was above 3%. The highest and lowest results from five measurements were discarded, after which, the average of the three remaining measurements was calculated for each site. All measurements were taken with the axial foot (opposite to the dominant foot). The thigh was measured in the supine position with the knee joint extended, whereas the lower leg was measured in the prone position with the knee joint extended and the foot hanging down over the edge of the bed (ankle-joint resting position). The order of the measurements was randomized for the lower leg, thigh, and each part of the body. Considering that changes in position during measurement may affect the values of the stiffness, all participants were instructed to rest for 10 min after identifying the muscle-tendon units and after changes in the lower leg and thigh position. All measurements were carried out by the same experienced physical therapist (R.S.).

### Reliability of measurements

The reliability of the musculotendon mechanical property measurements was examined in five healthy male university students (10 feet; mean age ± SD, 21 ± 0 years; height, 173.6 cm ± 2.2 cm; weight, 63.3 ± 3.9 kg) without any history of orthopedic disease using the same digital device. Repeat measurements were carried out between 2 days and 1 week. The intraclass correlation coefficient (ICC) (1, 3) was used to calculate reliability. The ICCs for the RF, VM, PT, MG, SOL, and AT were 0.802, 0.653, 0.968, 0.746, 0.774, and 0.835, respectively. Based on the criteria of Landis and Koch (24), reliability was considered substantial for an ICC of 0.61–0.80, which was taken to represent practical reliability.

### Statistical analysis

IBM SPSS Statistics version 28.0 (IBM Corp., Armonk, NY, USA) was used for the data analysis. Paired *t*-tests were performed to compare E2 and P4 concentrations and the stiffness of the muscles and tendons in the early follicular and early luteal phases, and Pearson's product-moment correlation coefficient to investigate correlations between the thigh and lower leg muscle and tendons. Cohen's *d* was used to calculate effect sizes as follows: trivial (0–0.19), small (0.20–0.49), medium (0.50–0.79), and large (>0.80) (25). Differences at the 5% level were considered to indicate statistical significance.

## Results

**Figure 4:**
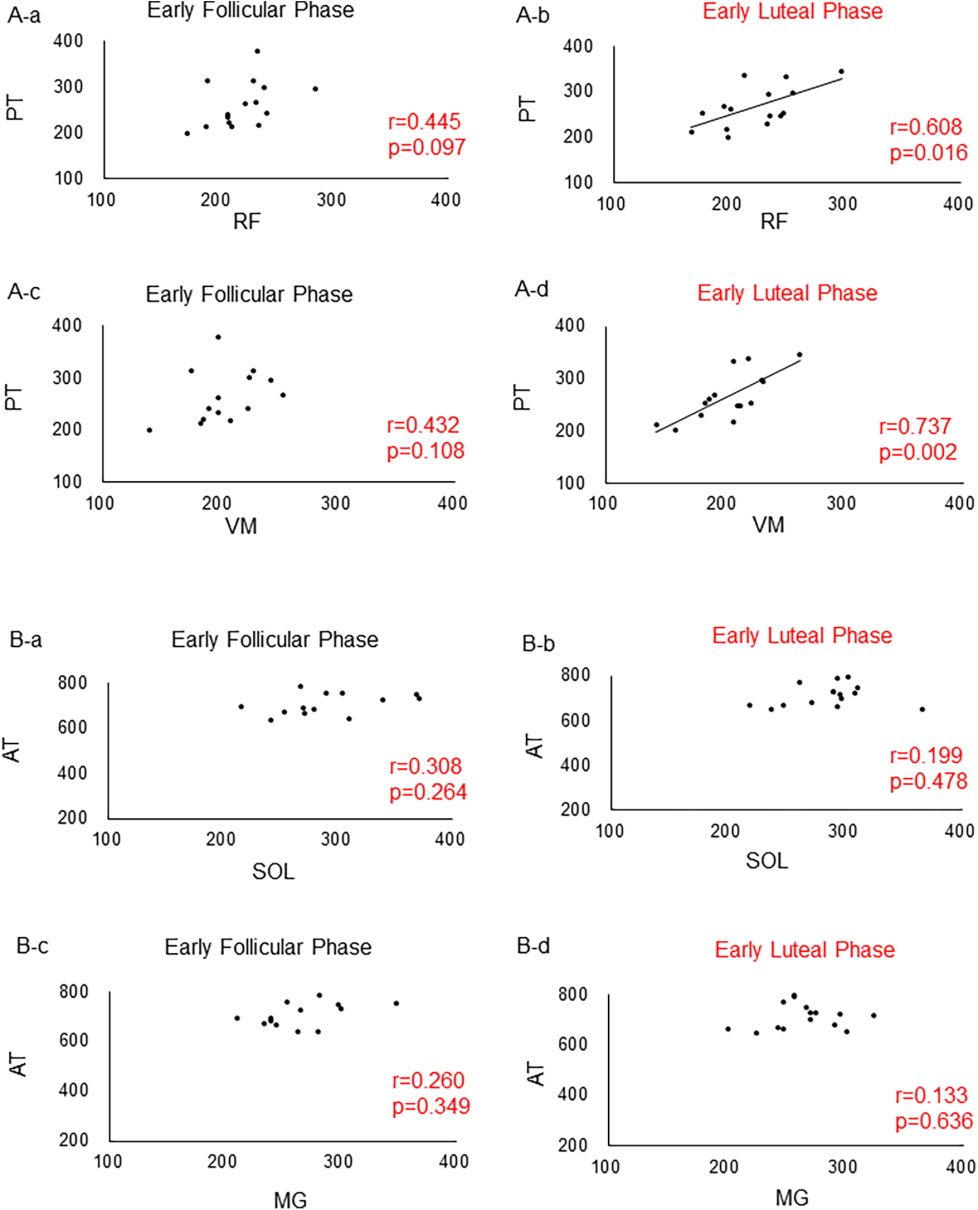
Correlations between the lower leg and thigh muscles and tendons. (**A**) Correlations between muscles and tendons in the thigh. (**A**) Between RF and PT in the early follicular phase. (**B**) Between RF and PT in the ovulatory phase. (**C**) Between VM and PT in the early follicular phase. (**D**) Between VM and PT in the ovulatory phase. (**B**) Correlations between muscles and tendons in the lower leg. (**A**) Between SOL and AT in the early follicular phase. (**B**) Between SOL and AT in the ovulatory phase. (**C**) Between MG and AT in the early follicular phase. (**D**) Between MG and AT in the ovulatory phase. RF, rectus femoris; VM, vastus medialis; PT, patellar tendon; SOL, soleus; MG, medial head of the gastrocnemius; AT, achilles tendon.

The results showed that the E2 concentration was significantly higher in the early luteal than in the early follicular phase (*p* = 0.017) (Table 1). However, no significant changes in the P4 concentration were observed between the early follicular and early luteal phases (*p* = 0.153) (Table 1). In addition, no significant difference in the stiffness of all muscles and tendons was found between the early follicular and early luteal phases (*p* > 0.05) (Table 1). No correlation was found between various muscles and tendon stiffness in the lower leg (Figure 4A). By contrast, during the early luteal phase, significant positive correlations were found between the RF and PT (*r* = 0.608, *p* = 0.016) and between the VM and PT (*r* = 0.737, *p* = 0.002) in the thigh (Figure 4B).

**Table 1:** Changes in estradiol and progesterone concentrations and stiffness during the menstrual cycle.

	Early follicular phase	Early luteal phase	*p*-values	Effect size
Estradiol [pg/ml]	1.44 ± 0.40	1.70 ± 0.40	0.017	0.66
Progesterone [pg/ml]	65.85 ± 38.94	82.73 ± 39.03	0.153	0.43
Stiffness[N/m]				
Rectus femoris	220.38 ± 26.20	222.84 ± 33.06	0.640	0.08
Vastus medialis	201.07 ± 29.29	203.27 ± 29.33	0.561	0.07
Patellar tendon	261.13 ± 48.55	266.89 ± 44.58	0.590	0.12
Medial head of gastrocnemius	263.84 ± 32.82	264.73 ± 30.04	0.834	0.03
Soleus muscle	290.53 ± 42.01	285.29 ± 34.09	0.500	0.13
Achilles tendon	720.07 ± 53.57	713.18 ± 47.82	0.696	0.13

Values are presented as mean ± standard deviation (*n* = 15).

RF, rectus femoris muscle; VM, vastus medialis muscle; PT, patellar tendon; SOL, soleus muscle; MG, medial head of gastrocnemius muscle; AT, achilles tendon.

## Discussion

The present study aimed to aimed to investigate changes in the stiffness of the thigh and lower leg muscles and tendons during the early follicular and early luteal phases of the menstrual cycle among female university students using a noninvasive digital palpitation device. To the best of our knowledge, no previous studies have provided a consistent view of changes in the stiffness of muscles and tendons during the menstrual cycle. Therefore, we believe that this constitutes the first study to investigate the relationships between the stiffness of muscles and tendons during the early follicular and early luteal phases of the menstrual cycle using a digital palpitation device.

No significant differences in stiffness of any muscles or tendons were found between the early follicular and early luteal phases. In previous study, reported that anterior knee laxity increased during the ovulatory compared with the early follicular phase in females with genu recurvatum (26). In addition, another previous study reported that an increase in the elasticity of the plantar fascia during the ovulatory phase increased postural sway and the risk of falls (27), other previous study found that strenuous exercise resulted in a rapid increase in collagen synthesis in human tendon and muscle the stiffness (28). Although methodologies differ and it is difficult to draw a general conclusion, these previous findings suggest that increased joint laxity in the knee and ankle joints during the ovulatory phase increases the musculotendinous load to compensate for tissue instability and stiffening. In addition, a previous study reported that E2 may affect the degradation of type I collagen by MMPs, which are degrading enzymes during the menstrual cycle (7). Therefore, the action of E2 may counteract this response to maintain joint stability. In addition, although a digital palpation device was used in the present study, previous studies (9, 11) have used a variety of methods to evaluate the mechanical properties of the muscle-tendon units. Furthermore, because opinions differ on issues such as changes in skeletal muscle strength during the menstrual cycle (29–31), further validation is needed, including evaluations of skeletal muscle strength and neural activation levels and verification among various evaluation methods.

In the present study, we found significant positive correlations between the stiffness from the RF and PT and between the VM and PT during the ovulatory phase. A previous study using the same palpation device reported that the stiffness of the VM and ST in the thigh muscle group was higher during the ovulatory than during the luteal phase; however, no such changes were observed for the VL or BF (12). These findings suggest that the quadriceps muscle and the medial side of the hamstrings (ST) may be affected during the ovulatory phase, when the E2 concentration is higher (12). However, a previous study involving females who had undergone reconstruction of the ACL reported no differences in muscle stiffness (ST and BF) on both sides during the early follicular and ovulatory phases (20). In addition, no periodic changes in the stiffness of the PL, TA, or MG were seen in the lower leg muscle group (14). Therefore, the degree of change in the mechanical properties of the muscle-tendon complex remains controversial. However, interestingly, significant correlations were observed only between the stiffness of the RF and PT and between the VM and PT during ovulation in this study. This site-specific change may provide insight into the mechanism of muscle-tendon injury. Therefore, further research on this issue is warranted.

This study has several limitations. First, the sample size was small. A larger sample size is needed to confirm the changes in the mechanical properties of the thigh and lower leg musculotendons during the early follicular and ovulatory phases, and to determine correlations between muscles and tendons. Second, this study does not take into account subcutaneous fat thickness, which may affect measurement results. Third, the measurement limb position (ankle joint position) was not strictly defined. Fourth, the MyotonPRO used in the present study measures only the surface layer of the muscle and a portion of that muscle at rest, and thus, may not capture changes in the entire muscle or deeper layers. In addition, it is unclear whether tendon stiffness by MyotonPRO truly reflects the condition of the muscle when it is contracting. MyotonePro primarily measures oscillation in the transverse direction, not necessarily axial stiffness like shear wave elastography or dynamometry/ultrasound techniques might. Therefore, it may be difficult to apply MyotonPRO measurements to human performance injury. Fifth, this study that the reliability was measured with male subjects, and therefore the reliability obtained with female subjects may be smaller due to possible influences of the menstrual cycle. Finally, although the basal body temperature method, salivary hormone concentration analysis, and an ovulation prediction kit were used to classify the menstrual cycle, whether the early luteal phase was strictly defined remains unclear. Future studies should use blood sampling for more appropriate monitoring of the menstrual cycle.

In conclusion, in the present study, no significant difference in muscle or tendon stiffness was observed in the anterior or lower posterior thigh regions between the early follicular and early luteal phases of menstruation. Only the anterior thigh region showed a significant positive correlation with the early luteal phases. These results suggest that the stiffness of the muscle-tendon units of the anterior and posterior thighs do not change between the early follicular and early luteal phases, and that tendons may be stiffer in those who have stiffer anterior thigh muscles during the early luteal phases.

## Data Availability

The raw data supporting the conclusions of this article will be made available by the authors, without undue reservation.
